# Synthesis, crystal structure and Hirshfeld surface analysis of di-μ_2_-iodido-bis­[(2,2′-bi­quinoline-κ^2^ *N*,*N*′)copper(I)]

**DOI:** 10.1107/S2056989023000634

**Published:** 2023-02-07

**Authors:** Ayalew W. Temesgen, Anton P. Novikov, Alexander G. Tskhovrebov, Ekaterina K. Kultyshkina, Tuan Anh Le

**Affiliations:** a Department of Chemistry, College of Natural and Computational Science, University of Gondar, Gondar 196, Ethiopia; b Peoples’ Friendship University of Russia (RUDN University), 6 Miklukho-Maklaya, St, 117198, Moscow, Russian Federation; c Frumkin Institute of Physical Chemistry and Electrochemistry, Russian Academy of Sciences, 31 Leninsky Prospekt bldg 4, 119071 Moscow, Russian Federation; d University of Science, Vietnam National University, Hanoi, 334 Nguyen Trai, Thanh Xuan, 100000, Hanoi, Vietnam; Universidad de Los Andes Mérida, Venezuela

**Keywords:** crystal structure, Hirshfeld surface analysis, π–π stacking, bi­quinoline, copper complex

## Abstract

The mol­ecular and crystal structures of the title compound, [Cu_2_I_2_(C_18_H_12_N_2_)_2_], were examined by single-crystal X-ray diffraction and Hirshfeld surface analysis. The Cu atom is coordinated in a distorted tetra­hedral geometry by two N atoms from the 2,2′-bi­quinoline ligands and the two μ_2_-bridging iodide ligands. The mol­ecules are in contact *via* π–π-stacking inter­actions. Hirshfeld surface analysis showed that the most important contributions to the inter­molecular inter­actions are H⋯H (39.7%), H⋯I/I⋯H (17.8%), C⋯H/H⋯C (17.5%), C⋯C (16.5%), N⋯C/C⋯N (3.9%) and N⋯H/H⋯N (3.5%).

## Abstract 2

In the layer structure of di-μ_2_-iodido-bis­[(2,2′-bi­quinoline-κ^2^ *N*,*N*′)copper(I)], π–π inter­actions provide conectivity within and between the layers.

## 1. Chemical context

Metal complexes with N-heterocyclic ligands find wide applications in various fields such as catalysis and medicine, among others (Delgado-Rebollo *et al.*, 2019 ▸; Novikov *et al.*, 2021 ▸; Fong, 2016 ▸; Artemjev *et al.*, 2022 ▸). Copper(I) bypiridine complexes are of inter­est because of their structural peculiarities, cuprophilic inter­actions, and important photochemical properties. Therefore, bypyridine-type systems are often the ligands of choice to explore new metal complexes with potentially useful properties (Ferraro *et al.*, 2022 ▸; Starosta *et al.*, 2012 ▸; Vatsadze *et al.*, 2010 ▸). 2,2′-Bi­quinoline is an important and widely employed di­imine ligand. The geometry of the resulting metal derivatives depends on the ligand and counter-ion, the metal:ligand ratio and the solvent and synthetic conditions. Here we report the preparation and structural characterization of a copper iodide complex with 2,2′-bi­quinoline. We used Hirshfeld surface analysis to estimate the contribution of non-covalent inter­actions to the crystal structure. 
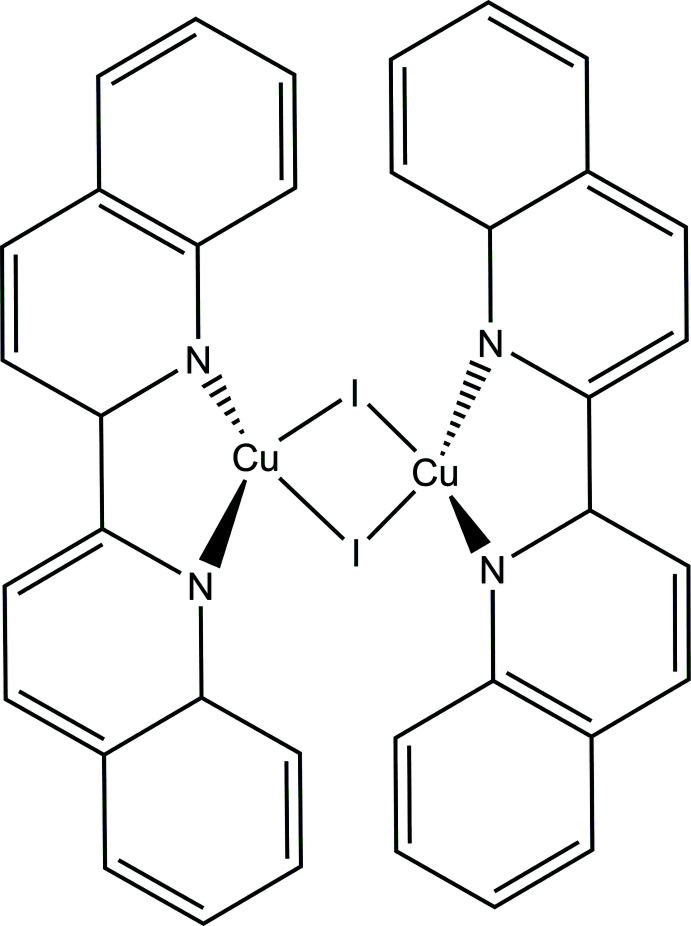


## 2. Structural commentary

**Figure 1:**
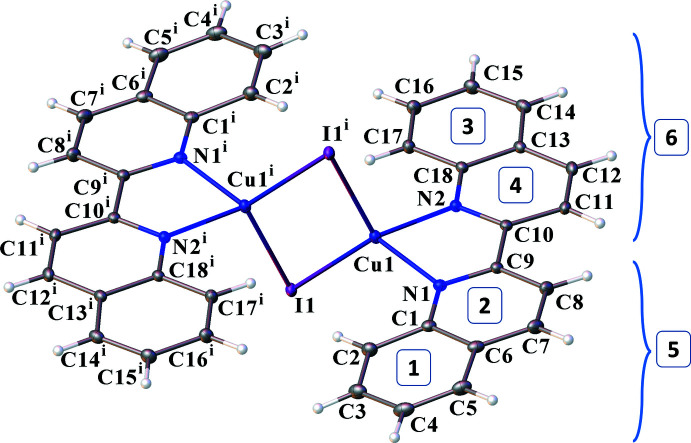
Mol­ecular structure of the title compound, including atom and ring labelling. Displacement ellipsoids are drawn at the 50% probability level. [Symmetry code: (i) −*x* + 1, −*y*, −*z* + 1.]

The title compound crystallizes in the centrosymmetric space group *P* 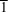 with one crystallographically independent mol­ecule in the unit cell. The mol­ecular structure is illustrated in Fig. 1 ▸. The Cu atom is coordinated in a distorted tetra­hedral geometry (Table 1 ▸) by two nitro­gen atoms from the 2,2′-bi­quinoline ligands and the two μ_2_-bridged iodide ligands. The Cu1—I1 and Cu1^i^—I1 distances [symmetry code: (i) −*x* + 1, −*y*, −*z* + 1] are 2.5734 (2) and 2.6487 (2) Å, which are close to the distances in similar compounds (Sun *et al.*, 2013 ▸; Starosta *et al.*, 2012 ▸) with a substituted quinoline ligand. The Cu—N distances of 2.0930 (13) and 2.0900 (14) Å are almost equal within standard uncertainty.

The quinoline fragments in the bi­quinoline ligand adopt, as expected, a planar geometry. The maximum and minimum deviations of the atoms from these planes are between −0.018 (2) and 0.026 (2) Å. The angle between the quinolines described by rings 1/2 (as defined in Fig. 1 ▸) is 5.08 (9)° and between 3/4 is 0.59 (8)°. Then, the quinoline formed by rings 1 and 2 (ring 5) makes an angle of 7.56 (5)° with the quinoline described by rings 3/4 (ring 6).

## 3. Supra­molecular features

**Figure 2:**
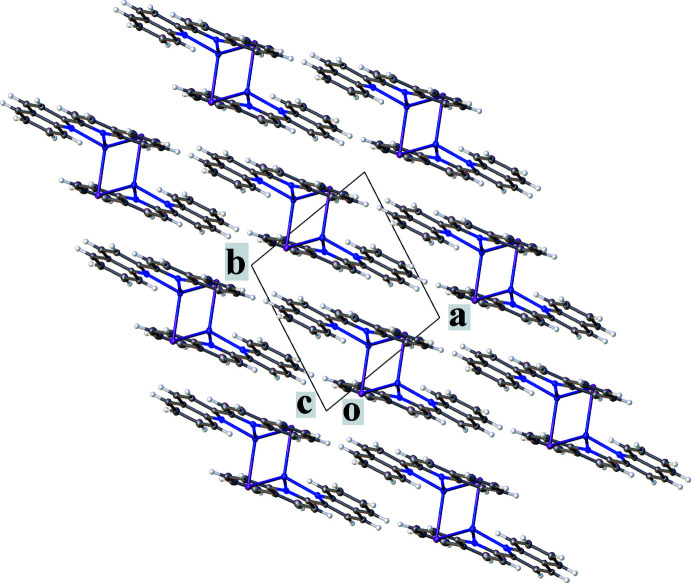
View along the *c* axis of the crystal packing of the title compound, showing the stacking of layers formed by the Cu complex.

The crystal packing is shown in Fig. 2 ▸, viewed down the c axis. Mol­ecules both within the layers and between them are connected by π–π-stacking inter­actions between six-membered rings of the quinoline rings. The π–π-stacking inter­action parameters are presented in Table 2 ▸. Ring 4, defined by N2/C18/C10–C13 in Fig. 1 ▸, participates in the shortest inter­actions. The contact with another ring 4, related by the symmetry operation −*x*, −*y* + 1, −*z* + 1, is perhaps the most efficient, based on the distance, the angle between the planes, and the shift between ring centroids.

## 4. Database survey

A search in the Cambridge Structural Database (CSD, Version 5.43, update of 2022; Groom *et al.*, 2016 ▸) showed only a few hits for bis­[(μ_2_-halogen)-2,2′-bi­quinoline-di-copper(I)]. We only found data for compounds with substituted quinoline rings in position-4 with carboxyl­ate fragments. All compounds crystallize in the triclinic space group *P* 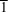 . In IRIVIP (Vatsadze *et al.*, 2010 ▸), *n*-hexyl carboxyl­ate groups are attached to the quinoline rings at position 4. In YIJFAA, YIJFEE, and YIJFII (Sun *et al.*, 2013 ▸), ethyl carboxyl­ate fragments are attached, and in PAYKIL (Starosta *et al.*, 2012 ▸), there are methyl carboxyl­ate fragments. In IRIVIP and YIJFAA, instead of the iodine atom, as in the title structure, there are chlorine atoms; in YIJFEE, there are bromine atoms. In other structures, the copper atoms are bonded through iodine atoms.

## 5. Hirshfeld surface analysis

**Figure 3:**
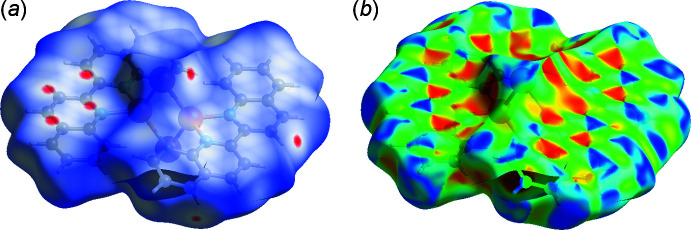
Hirshfeld surface mapped over (*a*) *d*_norm_ and (*b*) shape-index to visualize the inter­actions in the title compound.

**Figure 4:**
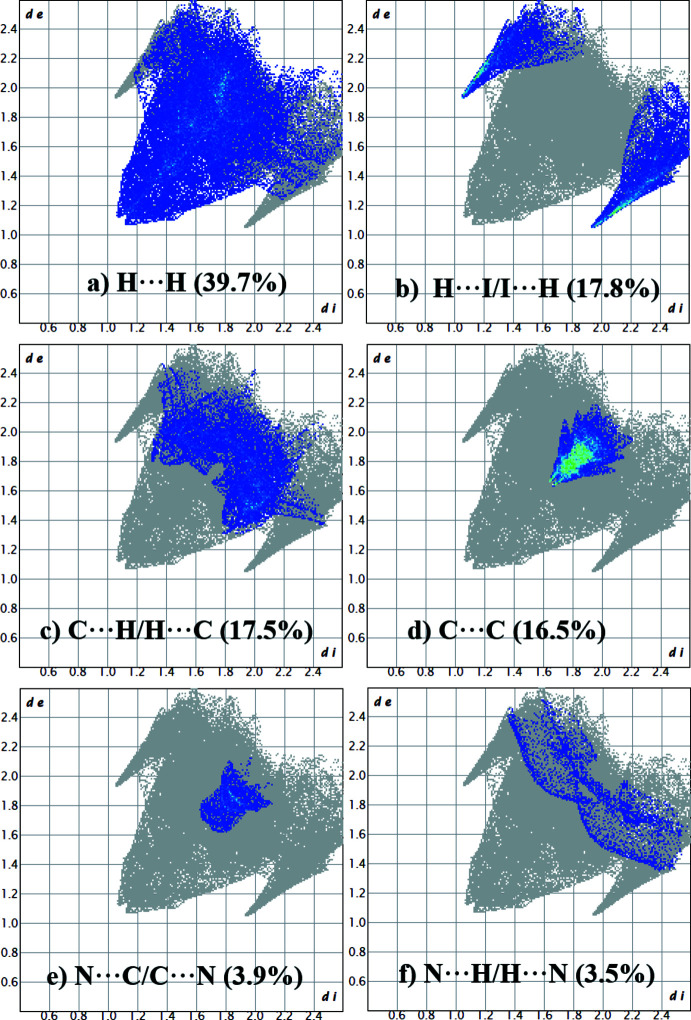
Two-dimensional fingerprint plots for the title compound divided into H⋯H (39.7%), H⋯I/I⋯H (17.8%), C⋯H/H⋯C (17.5%), C⋯C (16.5%), N⋯C/C⋯N (3.9%) and N⋯H/H⋯N (3.5%) inter­actions.

*Crystal Explorer21* was used to calculate the Hirshfeld surfaces and two-dimensional fingerprint plots (Spackman *et al.*, 2021 ▸). The donor–acceptor groups are visualized using a standard (high) surface resolution and *d*_norm_ surfaces are mapped over a fixed colour scale from −0.0579 (red) to 1.3919 (blue) a.u., as illustrated in Fig. 3 ▸(*a*). Red spots on the surface correspond to C⋯C and I⋯H inter­actions. The presence of π-stacking inter­actions is confirmed by the characteristic red and blue triangles on the shape-index surface [Fig. 3 ▸(*b*)]. Fingerprint plots of the most important non-covalent inter­actions for the title compound are shown in Fig. 4 ▸. The largest contribution to the crystal packing is made by contacts of the H⋯H type (39.7%). Then contacts of the H⋯I/I⋯H and C⋯H/H⋯C types make approximately equal contributions (17.8 and 17.5%, respectively). C⋯C inter­actions responsible for π-stacking contribute 16.5%. Contacts that contribute less than 1% are not shown in Fig. 4 ▸.

## 6. Synthesis and crystallization

The title compound was prepared by refluxing CuI with one equivalent of 2,2′-bi­quinoline in ethanol for 24 h. The compound precipitates as a purple solid in 87% yield. Found (%): C, 48.39; H, 2.71; N, 6.27. forC_36_H_24_Cu_2_I_2_N_4_. Calculated (%): C, 48.61; H, 2.64; N, 6.19.

## 7. Refinement

Crystal data, data collection and structure refinement details are summarized in Table 3 ▸. C-bound H atoms were placed at calculated positions (C—H = 0.95 Å) and refined using a riding model with [*U*_iso_(H) = 1.2*U*_eq_(C)].

## Supplementary Material

Crystal structure: contains datablock(s) I. DOI: 10.1107/S2056989023000634/dj2055sup1.cif

Structure factors: contains datablock(s) I. DOI: 10.1107/S2056989023000634/dj2055Isup2.hkl

CCDC reference: 2237760

Additional supporting information:  crystallographic information; 3D view; checkCIF report

## Figures and Tables

**Table 1:** Selected geometric parameters (Å, °)

I1—Cu1	2.5734 (2)	Cu1—N2	2.0900 (14)
I1—Cu1^i^	2.6487 (2)	Cu1—N1	2.0930 (13)
			
Cu1—I1—Cu1^i^	68.829 (8)	N2—Cu1—I1^i^	110.91 (4)
N2—Cu1—N1	79.28 (5)	N1—Cu1—I1^i^	106.99 (4)
N2—Cu1—I1	122.14 (4)	I1—Cu1—I1^i^	111.171 (8)
N1—Cu1—I1	122.34 (4)		

Symmetry code: (i) 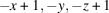 .

**Table 2:** π–π-stacking inter­action parameters (Å, °)

Ring 1	Ring No.	Ring 2	Ring No.	Angle	Centroid–centroid distance	Shift distance between ring centroids
C1–C6	1	C1–C6(−*x* + 1, −*y*, −*z* + 2)	1	0.000	3.874	1.459
C13–C18	3	N1/C1/C6–C9(−*x* + 1, −*y* + 1, −*z* + 1)	2	4.772	3.711	1.480
		N2/C18/C10–C13(−*x*, −*y* + 1, −*z* + 1)	4	0.590	3.665	1.602
N1/C1/C6–C9	2	N2/C18/C10–C13(−*x* + 1, −*y* + 1, −*z* + 1)	4	5.301	3.564	1.139
		C13–C18(−*x* + 1, −*y* + 1, −*z* + 1)	3	4.772	3.711	1.283
N2/C18/C10–C13	4	N2/C18/C10–C13(−*x*, −*y* + 1, −*z* + 1)	4	0.000	3.652	1.555
		C13–C18(−*x*, −*y* + 1, −*z* + 1)	3	0.590	3.665	1.579
		N1/C1/C6–C9(−*x* + 1, −*y* + 1, −*z* + 1)	2	5.301	3.564	1.068

**Table 3:** Experimental details

Crystal data	
Chemical formula	[Cu_2_I_2_(C_18_H_12_N_2_)_2_]
*M*_r_	893.49
Crystal system, space group	Triclinic, *P* 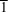
Temperature (K)	100
*a*, *b*, *c* (Å)	8.2032 (2), 9.4084 (3), 10.8312 (3)
α, β, γ (°)	70.9328 (8), 76.1237 (9), 74.2486 (9)
*V* (Å^3^)	749.84 (4)
*Z*	1
Radiation type	Mo *K*α
μ (mm^−1^)	3.51
Crystal size (mm)	0.12 × 0.10 × 0.06

Data collection	
Diffractometer	Bruker D8 QUEST PHOTON-III CCD
Absorption correction	Multi-scan (*SADABS*; Krause *et al.*, 2015 ▸)
*T*_min_, *T*_max_	0.656, 0.798
No. of measured, independent and observed [*I* > 2σ(*I*)] reflections	22231, 5464, 4875
*R*_int_	0.030
(sin θ/λ)_max_ (Å^−1^)	0.759

Refinement	
*R*[*F* ^2^ > 2σ(*F* ^2^)], *wR*(*F* ^2^), *S*	0.021, 0.050, 1.07
No. of reflections	5464
No. of parameters	200
H-atom treatment	H-atom parameters constrained
Δρ_max_, Δρ_min_ (e Å^−3^)	0.93, −1.00

Computer programs: *APEX3* (Bruker, 2018 ▸), *SAINT* (Bruker, 2013 ▸), *SHELXT* (Sheldrick, 2015*a* ▸), *SHELXL* (Sheldrick, 2015*b* ▸) and *SHELXTL* (Sheldrick, 2008 ▸).

## supplementary crystallographic information

### Crystal data

**Table:**

[Cu_2_I_2_(C_18_H_12_N_2_)_2_]	*Z* = 1
*M**_r_* = 893.49	*F*(000) = 432
Triclinic, *P*1	*D*_x_ = 1.979 Mg m^−^^3^
*a* = 8.2032 (2) Å	Mo *K*α radiation, λ = 0.71073 Å
*b* = 9.4084 (3) Å	Cell parameters from 9951 reflections
*c* = 10.8312 (3) Å	θ = 2.3–32.6°
α = 70.9328 (8)°	µ = 3.51 mm^−^^1^
β = 76.1237 (9)°	*T* = 100 K
γ = 74.2486 (9)°	Plate, red
*V* = 749.84 (4) Å^3^	0.12 × 0.10 × 0.06 mm

### Data collection

**Table:**

Bruker D8 QUEST PHOTON-III CCD diffractometer	4875 reflections with *I* > 2σ(*I*)
φ and ω scans	*R*_int_ = 0.030
Absorption correction: multi-scan (SADABS; Krause *et al.*, 2015)	θ_max_ = 32.6°, θ_min_ = 2.3°
*T*_min_ = 0.656, *T*_max_ = 0.798	*h* = −12→12
22231 measured reflections	*k* = −14→14
5464 independent reflections	*l* = −16→16

### Refinement

**Table:**

Refinement on *F*^2^	Secondary atom site location: difference Fourier map
Least-squares matrix: full	Hydrogen site location: inferred from neighbouring sites
*R*[*F*^2^ > 2σ(*F*^2^)] = 0.021	H-atom parameters constrained
*wR*(*F*^2^) = 0.050	*w* = 1/[σ^2^(*F*_o_^2^) + (0.0241*P*)^2^ + 0.2045*P*] where *P* = (*F*_o_^2^ + 2*F*_c_^2^)/3
*S* = 1.07	(Δ/σ)_max_ = 0.001
5464 reflections	Δρ_max_ = 0.93 e Å^−^^3^
200 parameters	Δρ_min_ = −1.00 e Å^−^^3^
0 restraints	Extinction correction: SHELXL, Fc^*^=kFc[1+0.001xFc^2^λ^3^/sin(2θ)]^-1/4^
Primary atom site location: difference Fourier map	Extinction coefficient: 0.00061 (6)

### Special details

**Table:**

Geometry. All esds (except the esd in the dihedral angle between two l.s. planes) are estimated using the full covariance matrix. The cell esds are taken into account individually in the estimation of esds in distances, angles and torsion angles; correlations between esds in cell parameters are only used when they are defined by crystal symmetry. An approximate (isotropic) treatment of cell esds is used for estimating esds involving l.s. planes.

### Fractional atomic coordinates and isotropic or equivalent isotropic displacement parameters (Å^2^)

**Table:**

	*x*	*y*	*z*	*U*_iso_*/*U*_eq_	
I1	0.72676 (2)	0.05497 (2)	0.36228 (2)	0.01463 (4)	
Cu1	0.47151 (3)	0.14895 (2)	0.52815 (2)	0.01482 (5)	
N1	0.50362 (17)	0.22489 (16)	0.68043 (14)	0.0137 (2)	
N2	0.32363 (17)	0.37275 (15)	0.48351 (13)	0.0126 (2)	
C1	0.5986 (2)	0.14322 (19)	0.77875 (16)	0.0149 (3)	
C2	0.7206 (2)	0.0086 (2)	0.76410 (18)	0.0188 (3)	
H2	0.7335	−0.0253	0.6881	0.023*	
C3	0.8205 (2)	−0.0730 (2)	0.86007 (19)	0.0225 (3)	
H3	0.9050	−0.1616	0.8486	0.027*	
C4	0.7991 (3)	−0.0268 (2)	0.97589 (19)	0.0235 (4)	
H4	0.8658	−0.0869	1.0430	0.028*	
C5	0.6831 (2)	0.1034 (2)	0.99178 (18)	0.0221 (3)	
H5	0.6688	0.1333	1.0701	0.027*	
C6	0.5837 (2)	0.1942 (2)	0.89164 (16)	0.0168 (3)	
C7	0.4734 (2)	0.3365 (2)	0.89624 (17)	0.0204 (3)	
H7	0.4603	0.3741	0.9702	0.024*	
C8	0.3849 (2)	0.4209 (2)	0.79434 (17)	0.0186 (3)	
H8	0.3134	0.5187	0.7954	0.022*	
C9	0.4017 (2)	0.35984 (18)	0.68682 (16)	0.0134 (3)	
C10	0.30656 (19)	0.44564 (18)	0.57445 (16)	0.0130 (3)	
C11	0.2064 (2)	0.59515 (18)	0.56565 (17)	0.0151 (3)	
H11	0.1989	0.6436	0.6318	0.018*	
C12	0.1199 (2)	0.67017 (18)	0.46130 (17)	0.0161 (3)	
H12	0.0505	0.7703	0.4552	0.019*	
C13	0.1348 (2)	0.59756 (18)	0.36296 (16)	0.0135 (3)	
C14	0.0488 (2)	0.6683 (2)	0.25249 (17)	0.0171 (3)	
H14	−0.0225	0.7681	0.2431	0.021*	
C15	0.0680 (2)	0.5933 (2)	0.15911 (17)	0.0183 (3)	
H15	0.0103	0.6413	0.0850	0.022*	
C16	0.1738 (2)	0.4440 (2)	0.17292 (17)	0.0181 (3)	
H16	0.1868	0.3931	0.1074	0.022*	
C17	0.2579 (2)	0.37192 (19)	0.27946 (17)	0.0162 (3)	
H17	0.3279	0.2717	0.2876	0.019*	
C18	0.23990 (19)	0.44731 (18)	0.37738 (16)	0.0129 (3)	

### Atomic displacement parameters (Å^2^)

**Table:**

	*U*^11^	*U*^22^	*U*^33^	*U*^12^	*U*^13^	*U*^23^
I1	0.01422 (5)	0.01364 (5)	0.01633 (6)	−0.00129 (3)	−0.00081 (3)	−0.00714 (4)
Cu1	0.01437 (9)	0.01477 (9)	0.01559 (10)	0.00062 (7)	−0.00375 (7)	−0.00671 (7)
N1	0.0120 (6)	0.0154 (6)	0.0140 (6)	−0.0033 (5)	−0.0023 (5)	−0.0041 (5)
N2	0.0117 (5)	0.0134 (6)	0.0131 (6)	−0.0013 (5)	−0.0023 (4)	−0.0049 (5)
C1	0.0130 (7)	0.0179 (7)	0.0145 (7)	−0.0057 (6)	−0.0020 (5)	−0.0035 (6)
C2	0.0187 (8)	0.0184 (7)	0.0188 (8)	−0.0028 (6)	−0.0069 (6)	−0.0028 (6)
C3	0.0211 (8)	0.0196 (8)	0.0249 (9)	−0.0041 (7)	−0.0097 (7)	0.0003 (7)
C4	0.0250 (9)	0.0243 (9)	0.0207 (8)	−0.0102 (7)	−0.0117 (7)	0.0042 (7)
C5	0.0252 (9)	0.0282 (9)	0.0150 (7)	−0.0111 (7)	−0.0076 (6)	−0.0013 (7)
C6	0.0149 (7)	0.0234 (8)	0.0133 (7)	−0.0081 (6)	−0.0018 (5)	−0.0036 (6)
C7	0.0183 (8)	0.0309 (9)	0.0160 (8)	−0.0060 (7)	−0.0015 (6)	−0.0121 (7)
C8	0.0170 (7)	0.0244 (8)	0.0175 (8)	−0.0024 (6)	−0.0030 (6)	−0.0113 (7)
C9	0.0113 (6)	0.0162 (7)	0.0138 (7)	−0.0027 (5)	−0.0015 (5)	−0.0059 (6)
C10	0.0103 (6)	0.0148 (6)	0.0145 (7)	−0.0032 (5)	−0.0009 (5)	−0.0053 (5)
C11	0.0147 (7)	0.0140 (6)	0.0186 (7)	−0.0033 (5)	−0.0016 (5)	−0.0077 (6)
C12	0.0152 (7)	0.0118 (6)	0.0206 (8)	−0.0023 (5)	−0.0009 (6)	−0.0055 (6)
C13	0.0116 (6)	0.0118 (6)	0.0159 (7)	−0.0019 (5)	−0.0022 (5)	−0.0026 (5)
C14	0.0137 (7)	0.0162 (7)	0.0181 (8)	−0.0016 (6)	−0.0033 (6)	−0.0012 (6)
C15	0.0175 (7)	0.0188 (7)	0.0166 (7)	−0.0013 (6)	−0.0058 (6)	−0.0023 (6)
C16	0.0175 (7)	0.0212 (8)	0.0164 (7)	−0.0011 (6)	−0.0043 (6)	−0.0077 (6)
C17	0.0152 (7)	0.0162 (7)	0.0180 (7)	−0.0006 (6)	−0.0036 (6)	−0.0072 (6)
C18	0.0104 (6)	0.0135 (6)	0.0145 (7)	−0.0021 (5)	−0.0016 (5)	−0.0041 (5)

### Geometric parameters (Å, º)

**Table:**

I1—Cu1	2.5734 (2)	C7—C8	1.369 (3)
I1—Cu1^i^	2.6487 (2)	C7—H7	0.9500
Cu1—N2	2.0900 (14)	C8—C9	1.422 (2)
Cu1—N1	2.0930 (13)	C8—H8	0.9500
Cu1—I1^i^	2.6487 (2)	C9—C10	1.488 (2)
Cu1—Cu1^i^	2.9520 (4)	C10—C11	1.409 (2)
N1—C9	1.330 (2)	C11—C12	1.367 (2)
N1—C1	1.367 (2)	C11—H11	0.9500
N2—C10	1.3354 (19)	C12—C13	1.409 (2)
N2—C18	1.369 (2)	C12—H12	0.9500
C1—C2	1.414 (2)	C13—C14	1.415 (2)
C1—C6	1.421 (2)	C13—C18	1.423 (2)
C2—C3	1.374 (2)	C14—C15	1.369 (2)
C2—H2	0.9500	C14—H14	0.9500
C3—C4	1.415 (3)	C15—C16	1.418 (2)
C3—H3	0.9500	C15—H15	0.9500
C4—C5	1.365 (3)	C16—C17	1.372 (2)
C4—H4	0.9500	C16—H16	0.9500
C5—C6	1.418 (2)	C17—C18	1.418 (2)
C5—H5	0.9500	C17—H17	0.9500
C6—C7	1.406 (3)		
			
Cu1—I1—Cu1^i^	68.829 (8)	C8—C7—H7	119.9
N2—Cu1—N1	79.28 (5)	C6—C7—H7	119.9
N2—Cu1—I1	122.14 (4)	C7—C8—C9	118.99 (16)
N1—Cu1—I1	122.34 (4)	C7—C8—H8	120.5
N2—Cu1—I1^i^	110.91 (4)	C9—C8—H8	120.5
N1—Cu1—I1^i^	106.99 (4)	N1—C9—C8	122.17 (15)
I1—Cu1—I1^i^	111.171 (8)	N1—C9—C10	116.58 (13)
N2—Cu1—Cu1^i^	141.62 (4)	C8—C9—C10	121.24 (15)
N1—Cu1—Cu1^i^	136.76 (4)	N2—C10—C11	122.82 (15)
I1—Cu1—Cu1^i^	56.791 (7)	N2—C10—C9	115.92 (14)
I1^i^—Cu1—Cu1^i^	54.380 (7)	C11—C10—C9	121.26 (14)
C9—N1—C1	119.18 (14)	C12—C11—C10	119.63 (14)
C9—N1—Cu1	113.35 (11)	C12—C11—H11	120.2
C1—N1—Cu1	126.92 (11)	C10—C11—H11	120.2
C10—N2—C18	118.23 (14)	C11—C12—C13	119.38 (15)
C10—N2—Cu1	113.89 (11)	C11—C12—H12	120.3
C18—N2—Cu1	127.82 (10)	C13—C12—H12	120.3
N1—C1—C2	118.85 (15)	C12—C13—C14	122.40 (15)
N1—C1—C6	121.75 (15)	C12—C13—C18	117.93 (15)
C2—C1—C6	119.33 (16)	C14—C13—C18	119.67 (14)
C3—C2—C1	119.82 (17)	C15—C14—C13	120.22 (16)
C3—C2—H2	120.1	C15—C14—H14	119.9
C1—C2—H2	120.1	C13—C14—H14	119.9
C2—C3—C4	120.78 (18)	C14—C15—C16	120.11 (16)
C2—C3—H3	119.6	C14—C15—H15	119.9
C4—C3—H3	119.6	C16—C15—H15	119.9
C5—C4—C3	120.43 (17)	C17—C16—C15	121.04 (15)
C5—C4—H4	119.8	C17—C16—H16	119.5
C3—C4—H4	119.8	C15—C16—H16	119.5
C4—C5—C6	120.13 (17)	C16—C17—C18	119.85 (15)
C4—C5—H5	119.9	C16—C17—H17	120.1
C6—C5—H5	119.9	C18—C17—H17	120.1
C7—C6—C5	122.99 (16)	N2—C18—C17	118.90 (14)
C7—C6—C1	117.63 (16)	N2—C18—C13	122.00 (14)
C5—C6—C1	119.34 (17)	C17—C18—C13	119.10 (15)
C8—C7—C6	120.15 (15)		
			
C9—N1—C1—C2	−173.05 (15)	C18—N2—C10—C9	−179.42 (13)
Cu1—N1—C1—C2	16.1 (2)	Cu1—N2—C10—C9	3.07 (17)
C9—N1—C1—C6	3.8 (2)	N1—C9—C10—N2	4.8 (2)
Cu1—N1—C1—C6	−166.98 (11)	C8—C9—C10—N2	−175.83 (14)
N1—C1—C2—C3	178.35 (16)	N1—C9—C10—C11	−174.64 (14)
C6—C1—C2—C3	1.4 (3)	C8—C9—C10—C11	4.7 (2)
C1—C2—C3—C4	2.0 (3)	N2—C10—C11—C12	0.9 (2)
C2—C3—C4—C5	−2.6 (3)	C9—C10—C11—C12	−179.63 (15)
C3—C4—C5—C6	−0.4 (3)	C10—C11—C12—C13	−1.0 (2)
C4—C5—C6—C7	−174.13 (17)	C11—C12—C13—C14	179.90 (16)
C4—C5—C6—C1	3.8 (3)	C11—C12—C13—C18	0.2 (2)
N1—C1—C6—C7	−3.1 (2)	C12—C13—C14—C15	179.67 (15)
C2—C1—C6—C7	173.76 (16)	C18—C13—C14—C15	−0.7 (2)
N1—C1—C6—C5	178.82 (15)	C13—C14—C15—C16	0.2 (3)
C2—C1—C6—C5	−4.3 (2)	C14—C15—C16—C17	0.3 (3)
C5—C6—C7—C8	177.99 (17)	C15—C16—C17—C18	−0.3 (3)
C1—C6—C7—C8	0.0 (2)	C10—N2—C18—C17	179.51 (14)
C6—C7—C8—C9	2.2 (3)	Cu1—N2—C18—C17	−3.4 (2)
C1—N1—C9—C8	−1.5 (2)	C10—N2—C18—C13	−0.9 (2)
Cu1—N1—C9—C8	170.55 (12)	Cu1—N2—C18—C13	176.23 (11)
C1—N1—C9—C10	177.85 (13)	C16—C17—C18—N2	179.42 (15)
Cu1—N1—C9—C10	−10.14 (17)	C16—C17—C18—C13	−0.2 (2)
C7—C8—C9—N1	−1.6 (3)	C12—C13—C18—N2	0.7 (2)
C7—C8—C9—C10	179.14 (15)	C14—C13—C18—N2	−178.92 (15)
C18—N2—C10—C11	0.1 (2)	C12—C13—C18—C17	−179.65 (15)
Cu1—N2—C10—C11	−177.45 (12)	C14—C13—C18—C17	0.7 (2)

Symmetry code: (i) −*x*+1, −*y*, −*z*+1.
